# Physical activity, sedentary behaviors, and breakfast eating as factors influencing BMI in Saudi students, aged 10 to 15 years

**DOI:** 10.1080/07853890.2022.2077429

**Published:** 2022-05-20

**Authors:** Mohamed Ahmed Said, Mohammed Shaab Alibrahim

**Affiliations:** a Department of Physical Education, College of Education, King Faisal University, Al-Ahsa, Saudi Arabia; b Higher Institute of Sport and Physical Education of Kef, Tunisia

**Keywords:** Children, adolescents, body composition, energy expenditure, energy intake, lifestyle behaviours, overweight, obesity

## Abstract

**Background:**

Eating less and moving more are the simplest and most common strategies to combat excessive weight. Several other lifestyle factors can also contribute to maintaining a healthy weight.

**Objectives:**

The present study examined the effects of breakfast frequency, duration and quality of sleep, daily physical activity, sedentary behaviours, and school transportation on the BMI of Saudi students, aged 10–15 years.

**Materials and Methods:**

This study included 981 students (240 girls (24.46%) [66 children (27.5%) and 174 adolescents (72.5%)] and 741 boys (75.54%) [441 children (59.51%) and 300 adolescents (40.49%)]). For each participant, height, weight, and body composition were obtained using bioelectric impedance analysis. A questionnaire focussing on lifestyle behaviours over the last seven days was also completed by each student. Multiple comparisons were performed to test for significant differences between the groups, stratified by sex, age, and BMI. Stepwise multiple regression analysis was used to determine the variables that significantly affected BMI.

**Results:**

The overall prevalence of overweight and obesity in boys was 12.82% and 25.1%, and in girls, 10.42% and 5.42%, respectively. Most participants used cars or buses as transportation to and from school (100% of girls and 83% of boys). Breakfast was skipped mainly by male participants. Boys tended to sleep more than girls during school days (488.90 ± 74.33 vs. 467.76 ± 78.75 min. night^−1^). They were more active (2.58 ± 0.69 vs. 2.34 ± 0.82), used laptops more frequently (2.46 ± 1.51 vs. 1.90 ± 1.63), and played more video games (3.12 ± 1.43 vs. 1.2875 ± 1.36) than girls. However, girls were less sedentary (2.63 ± 0.76 vs. 2.9±.79), watched more TV (3.65 ± 1.155 vs. 2.73 ± 1.48) and used their smartphones more (3.6625 ± 1.3 vs. 3.28 ± 1.44) than boys.

**Conclusions:**

This study revealed significant associations between BMI and breakfast intake, physical activities, and sedentary behaviours. Of these, using laptops and playing video games were the key sedentary activities that influenced BMI.

**Key messages**
1. A significantly higher prevalence of overweight and obesity was noted among boys compared to girls in private school students, aged 10–15 years, in Al-Ahsa governorate, Eastern Province of Saudi Arabia.
2. Boys slept more than girls on school days, and they were more active, used laptops more frequently, and played more video games than girls. Girls were less sedentary, watched television more often, and used smartphones more frequently than boys.
3. Among private school students aged 10–15 years in Al-Ahsa governorate, Eastern Province of Saudi Arabia, BMI was significantly associated with breakfast intake frequency, physical activity, and sedentary behaviours, among which using laptops and playing video games were the sedentary activities that most influenced BMI.

## 1. Background

Non-pharmacological prevention and treatment of overweight and obesity are based on creating an imbalance between daily energy intake and expenditure. Eating less and moving more is the simplest and most common strategy to create this imbalance. Hill [1] stated that to lose weight, it is essential to decrease energy intake and increase energy output, and not just one or the other. Another method for reducing weight is by limiting the consumption of energy-dense foods and encouraging the consumption of low-energy-density, nutrient-rich substances [2]. Relevant studies have asserted that to maintain a healthy weight, maintaining an excessive degree of energy expenditure may be a more viable method for most people than prohibiting the consumption of meals to satisfy a low degree of energy intake [1].

Physical activity (PA), whether structured or unstructured, is the largest modifiable component of the energy expenditure part of the energy balance equation [3,4]. Therefore, increasing PA has the potential to increase energy expenditure, thus improving fat loss and maintaining healthy body weight. A previous study reported that compared to their inactive counterparts, physically active youths exhibit a lower proportion of body fat, harder bones, stronger muscles, and reduced risks of cardiometabolic diseases [5]. Gutin et al. [6] in their cross-sectional study of 421 high school students reported that those who indulged in higher amounts of moderate and vigorous PA were more likely to have a higher index of cardiometabolic aptitude and a low body fat percentage. Additionally, participants with improved cardiorespiratory fitness demonstrated a healthy cardiometabolic profile and had significantly lower risks for cardiometabolic diseases [6].

PA is also one of the factors most associated with the overall health-related quality of life. The impact of body mass index (BMI) on quality of life is indisputable. Evidence has confirmed that those with normal weight have the significantly higher health-related quality of life scores than those who are obese [7]. Consequently, the American Physical Activity Guidelines [5] recommended that school-aged children and adolescents, aged 6–17 years, should practice moderate to vigorous PA for at least 1 h each day to combat overweight and obesity [5]. However, studies have observed that most students do not reach the recommended amount of PA. Moreover, children and adolescents today are around 15% less fit than their parents when the parents were the same age [8].

With advances in technology, electronic devices have increasingly attracted the attention of children and adolescents, which in turn, has increased the level of sedentary activities, such as handheld devices and video game consoles. The increased time spent on electronics and screen time has become a potential factor that limits energy expenditure [9]. Thus, current guidelines call for limiting daily sedentary screen time to two hours or less in children and adolescents [3]. Wells et al. [10] found that both short sleep duration and increased television watching were associated with greater fat mass, excess body weight, and higher blood pressure in participants, aged 10–12 years. Bartosiewicz et al. [9] also noted that among young people, the amount of sleep, gaming, watching movies, and using smartphones seriously impacted their body weight and body composition. These authors recommended incorporating psychological and emotional aspects, daily PA, and the support of health organisations as solutions to promote good eating habits in children, adolescents, and their parents. Given that school-aged students spend around half of their daily time in school, where they consume at least one-third of their daily calories, a school is an ideal place for implementing strategies for preventing and combating obesity and its comorbidities [4].

In Al-Ahsa, the largest governorate in the Eastern Province of Saudi Arabia is a region of dry tropical climate, which is hot in summer and windy in winter. These environmental factors may limit physical activities, especially outdoors. In addition, the considerable socioeconomic development in Saudi Arabia, over the past four decades, has resulted in a significant increase in sedentary behaviours and physical inactivity, among children and adolescents. As a result, Saudi Arabia is among the countries with the highest prevalence rates of obesity and overweight. This study aimed to determine the prevalence of overweight and obesity among Saudi students, aged 10–15 years, in the Al-Ahsa governorate and assess the association between BMI and the level of daily PA, sedentary behaviours such as time spent watching television, and using new media (tablets, laptops, video game consoles, and smartphones), sleep quality and duration, breakfast intake frequency, and school transportation method.

## 2. Material and methods

### 2.1. Sample size

There are 22 private schools in the Al-Ahsa governorate. Of these, 12,594 students (6,394 boys and 6,200 girls) were enrolled. Eight of the 22 schools were randomly chosen, with two schools selected from each geographic area (east, west, north, and south). In each school, two classes from grades 4 to 8 were randomly selected. Assuming a prevalence (p) of 50%, with a required confidence level of 99%, a margin of error (e) of 4%, and an associated *z*-score of 2.576, the sample size (*n*) was calculated according to the formula: *n* = [z2 × p × (1 – p)/e2]/[1 + (z2 × p × (1 – p)/(e2 × N))] [11]. Considering a dropout rate of 20%, a minimum of 1150 students is required to participate. Almost all students from the 80 selected classes were invited to participate in this study, and 1780 requests were sent to parents. In total, 1238 positive responses (417 girls and 821 boys) were received.

#### 2.1.1. Exclusion criteria

Students aged less than 10 years or over 15 years, BMI greater than 35 kg.*m*^−2^, any amputation of any part of the body, an acute or chronic health condition, and failure to complete one or more stages of the study were excluded from this study. The lack of written parental consent for children to participate in our study was an exclusion criterion.

### 2.2. Participants

Following body weight, height, and body composition measurements, 188 students (134 girls and 54 boys) were excluded from the study due to incorrect responses or not completing all tests, of these, 25 were excluded due to excess weight (3 girls and 22 boys), and 44 due to a lack of interest (40 girls and four boys). A total of 981 students (240 girls and 741 boys) completed all stages of the study and were included in the study analysis. The data were entered into the anthropometric calculator V.3.1 of the World Health Organisation (Geneva) software Anthro plus V.1.0.4 [12]. Participants were classified according to BMI, age, and sex into four categories: (1) underweight (UW; BMI-for-age less than the 5th percentile), (2) normal weight (NW; BMI-for-age between the 5th percentile and less than the 85th percentile), (3) overweight (OW; BMI-for-age between the 85th percentile and less than the 95th percentile), and (4) obese (OB; BMI for age equal to or greater than the 95th percentile).

### 2.3. Procedures

Ethical approval was obtained from the Ethics Committee of the Deanship of Scientific Research, King Faisal University (Ref. No. KFU-REC-2021-OCT-EA00019). All parents were informed of the objectives, study details, and possible outcomes. Parents who agree to have their children participate in the study signed an informed consent form. The validity and reliability of the self-administered questionnaire were verified in a pre-test conducted by five life science experts and 40 students who were not part of the study sample. All adjustments proposed by the experts were implemented to make the language clearer and easier to understand. The Cronbach’s alpha test of reliability was satisfactory (*α* = 0.78). The verified questionnaire was distributed to all students, and height, body weight, and body composition were measured for each participant.

### 2.4. Study outcomes

#### 2.4.1. Anthropometry

For each participant, height was measured to the nearest 0.1 cm using a stadiometer (Holtain, Crymych, Wales, UK). Body weight and body composition were estimated by bioelectric impedance analysis using a Tanita segmental body composition monitor (FitScan BC-601, Japan). All measurements were performed according to the manufacturer’s guidelines with minimal clothing, clean feet, and consistent hydration conditions. Height, age, and sex were manually entered into the bioelectrical impedance analyser and weight, body fat percentage, and BMI were recorded. Fat mass was calculated as weight (kg) × body fat percentage, and fat-free mass was calculated as weight (kg) – fat mass (kg).

#### 2.4.2. Questionnaire

The student questionnaire focussed on physical activities and sedentary lifestyle-related behaviours, sleep habits, use of electronic devices, breakfast intake frequency, and transportation to and from school. It consisted of four parts and took approximately 25 min to complete. The first part collected demographic data (age, nationality, and sex). The second part consisted of the Physical Activity Questionnaire for Older Children (PAQ-C), a self-administered, 7-day recall instrument intended to provide general reporting of PA levels throughout the elementary school year for students in grades four to eight [13].

To ensure participants understood the questions, the English version of the PAQ-C was translated and adapted to the Saudi context following the recommendations of Beaton et al. [14]. The questionnaire was first translated into Arabic by two bilingual translators and a standardised version was extracted. This version was then back translated into English by two English speakers of Arab origin, and both versions (Arabic and English) were reviewed by five bilingual lifestyle experts. The necessary adjustments requested by the experts were made. Finally, the reliability of the Arabic version was examined separately and as a part of the overall questionnaire. Cronbach's alpha for the PAQ-C was 0.788.

The PAQ-C consists of 10 items, nine of which are used to provide a summary PA score. The tenth item assessed whether endogenous or exogenous factors prevented the child from regularly practising PA during the past week. The first nine items are scored on a five-point scale, with the lowest activity response of 1 and the highest activity response of 5. The average score made it possible to classify the participants into five categories: very sedentary, sedentary, moderately active, active, and very active. In addition, individuals were classified as “sedentary” if the average score was less than 3, and “active” if the average score was equal to or greater than 3.

The third part of the questionnaire asked about sedentary behaviours using the NSW School Physical Activity and Nutrition Survey [15]. It contains five items on sedentary activities involving the use of technological devices and the Internet (iPad, tablet, computer, or smartphone), watching television, movies, or programs on the Internet, and playing games (including on a computer, game console, smartphone, or iPad). Students responded with 0 = not at all, 1 = less than 30 min per day, 2 = 30 min–1 h per day, 3 = around 1–2 h per day, 4 = around 2–4 h per day, and 5 = more than 4 h per day. The average score for sedentary activities during the week and on the weekend was calculated by the sum of the score of each item and dividing it by the total number of items. The recommended duration of sedentary activity is 2 h or less per day. More than 2 h per day is considered high sedentary activity [16].

The fourth part of the questionnaire examined the children’s sleeping behaviour and breakfast intake frequency during the school week and on the weekend. The frequency of breakfast consumption, the average duration of sleep, and quality of sleep, were assessed with the following questions: How often did you eat breakfast in the last school week? How many times did you have breakfast last weekend? What time did you go to bed during the previous school week? What time did you go to bed the previous weekend? What time did you wake up in the last school week? What time did you wake up the previous weekend? During the past seven days, did you experience any night of bad sleep? Do you have trouble waking up? In the Saudi context, the days of the week are from Sunday to Thursday, and the weekend includes Friday and Saturday.

### 2.5. Data analysis

BMI for age, sex, and percentiles were calculated using the anthropometric calculator from WHO Anthro plus V.1.0.4, and the recommended cut-offs for underweight, normal weight, overweight, and obesity were used [17]. These four body size categories were set as the dependent variables. The independent variables were school transportation, breakfast intake frequency, sleep pattern and quality, PA, and sedentary behaviours. Statistical analysis was performed using SPSS V.26 (IBM, Armonk, NY, USA). Descriptive data were summarised as means and standard deviations or proportions for the total population. Male, female, and BMI subgroups were used to describe the characteristics of the sample. Differences in school transportation, breakfast intake frequency, and sleep quality, stratified by sex and age, were tested using chi-square tests. *T*-tests and one-way ANOVA analyses were used to test for significant differences between the groups, stratified by sex, age, and BMI for all continuous variables. The Bonferroni post hoc test was used to test between-group differences. The *p*-values were set to .05 for single comparisons and .01 for multiple comparisons to control the probability of type I errors (Bonferroni correction). The linearity of the predictor variables was tested using scatterplots. The normality and homoscedasticity of the residuals were also verified. The independent variables were checked for multicollinearity using variance inflation factor (VIF) values less than 5. Stepwise multiple regression analysis was used to determine the variables that significantly affected BMI. To avoid the confounding effect, two models were run, one with the sedentary behaviour score and the other with sedentary activity levels, and the results were reported as unstandardised beta coefficients (β and standard error) and R-squared.

## 3. Results

### 3.1. Anthropometric characteristics

**Figure 1.**
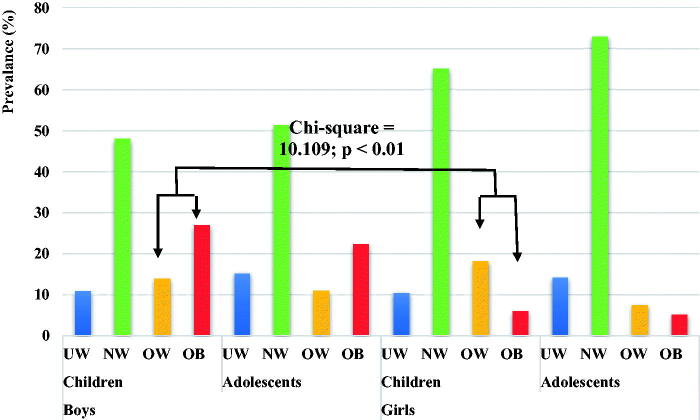
The prevalence of underweight (UW), normal weight (NW), overweight (OW), and obesity (OB) in students in private schools in the Al-Ahsa governorate, Saudi Arabia, stratified by sex, age, and BMI.

A total of 981 students completed all the parts of the study. Of these, 240 were girls (24.46%) aged 13.41 ± 1.17 years, comprising 66 children (11.92 ± 0.27 years; 27.5%) and 174 adolescents (13.98 ± 0.83 years; 72.5%), and 741 were boys (75.54%), aged 12.26 ± 1.53 years, comprising 441 children (11.58 ± 0.82 years; 59.51%) and 300 adolescents (13.82 ± 0.85 years; 40.49%). Referring to the sex-specific BMI-for-age percentile charts [10], the overall prevalence of overweight and obesity in boys was 12.82% and 25.1% and in girls 10.42% and 5.42%, respectively. The values relating to UW, NW, OW, and OB in boys were 12.82% (children: 11.11%; adolescents: 15.33%), 49.26% (children: 47.85%; adolescents: 51.33%%), 12.82% (children: 14.06%; adolescents: 11%), and 25.36% (children: 26.97%; adolescents: 22.33%), and in girls 13.33% (children: 10.6%; adolescents: 14.37%), 70.83% (children: 65.15%; adolescents: 72.99%), 10.42% (children: 18.18%; adolescents: 7.47%), and 5.42% (children: 6.06%; adolescents: 5.17%), respectively. The chi-square test revealed a significant difference in the prevalence of overweight and obesity in prepubescent boys compared to their female counterparts (*K*^2^ = 10.109; *p* < .01; Figure 1). Important differences, but not statistically ​significant, were noted in the remaining comparisons between groups of boys and girls. Significant prevalence on the side of girls was also noted in all parameters except BMI (*p* < .05, fat-free mass; *p* < .001 for the rest). However, for boys, the BMI was significantly lower in children than in adolescents (*p* < .008); The other variables except for body fat percentage were significantly lower in prepubescent boys than in the other three groups (*p* < .001 for all). The body fat percentage was higher in girls than in boys. Adolescent boys had the highest fat-free mass (*p* < .001 for all). No significant differences were noted between the two groups of girls stratified by age (Table 1).

**Table 1.** Anthropometric characteristics of students in private schools in the Al-Ahsa governorate, Saudi Arabia, stratified by sex and age.

		Height (cm)	Weight (kg)	BMI (kg.m^−2^)	PBF (%)	FM (kg)	FFM (kg)
Boys	Total (*N* = 741)	145.74 ± 12.74	41.85 ± 13.86	19.37 ± 4.68	21.07 ± 6.04	9.48 ± 5.79	32.37 ± 8.64
	Children (*N* = 441)	139.23 ± 8.67 b, c, d	37.22 ± 11.59 b, c, d	18.97 ± 4.67 b	20.88 ± 6.19 c, d	8.4 ± 5.20 b, c, d	28.83 ± 6.72 b, c, d
	Adolescents (*N* = 300)	155.30 ± 11.71 a	48.65 ± 14.14 a, c	19.97 ± 4.63 a	21.36 ± 5.79 c, d	11.07 ± 6.23 a	37.58 ± 8.52 a, c, d
Girls	Total (*N* = 240)	154.78 ± 7.18 ***	45.49 ± 8.63 ***	18.97 ± 3.33	24.83 ± 6.59 ***	11.66 ± 5.07 ***	33.83 ± 4.9 *
	Children (*N* = 66)	152.30 ± 6.37 a	44.17 ± 7.63 a, b	19.03 ± 3.23	24.24 ± 6.24 a, b	11.05 ± 4.56 a	33.12 ± 4.14 a, b
	Adolescents (*N* = 174)	155.72 ± 7.27 a	45.99 ± 8.96 a	18.95 ± 3.38	25.05 ± 6.73 a, b	11.9 ± 5.25 a	34.1 ± 5.14 a, b
Total children (*N* = 507)		140.93 ± 9.49	38.1286 ± 11.38699	18.9690 ± 4.50351	21.3168 ± 6.30059	8.7415 ± 5.19844	29.3874 ± 6.60133
Total adolescents (*N* = 474)		155.45 ± 10.3‴	47.6768 ± 12.54448‴	19.5926 ± 4.24001′	22.7149 ± 6.39794ʺ	11.3738 ± 5.89363‴	36.3038 ± 7.63723‴

Note:

Data represented as means ± standard deviations. a, b, c, d differs from prepubescent boys, adolescent boys, prepubescent girls, and adolescent girls, respectively, as determined by ANOVA analysis. *p < .05, *** < .001 differs from boys using *t*-test for independent samples. ′*p* < .05, ʺ*p* < .01, ′ʺ< .001 differs from children using *t*-test for independent samples. BMI: body mass index; BFP: percentage body fat; FM: fat mass; FFM: fat-free mass.

### 3.2. Physical activity and sedentary lifestyle–related characteristics

Table 2 shows the number and proportion of participants, stratified by sex and age category, for school transportation, breakfast intake, and sleep quality. The chi-square calculation demonstrated significant differences between boys and girls in the use of cars, buses, or walking to and from school (*χ*^2^ = 56.296; *p* < .001) as well as in breakfast intake on weekdays (*χ*^2^ = 37.592; *p* < .001) and the quality of sleep (*χ*^2^ = 10.188; *p* < .01). All the girls used cars (85.58%) or buses (14.42%) as transportation for school, 67.08% ate breakfast on three or more weekdays, and 48% slept well. Of boys, 17% walked to school and the rest used cars (62.48%) or buses (20.51%). Boys were less likely to have breakfast, with 59.38% eating breakfast on three weekdays or fewer and 72.2% having difficulty sleeping or waking up.

**Table 2.** Comparing school transportation, weekday and weekend breakfast frequency, and sleep quality among students in private schools in Al-Ahsa governorate, Saudi Arabia, stratified by sex and age.

			Boys			Girls			*P* value
			Total (*N* = 741)	Children (*N* = 441)	Adolescents (*N* = 300)	Total (*N* = 240)	Children (*N* = 66)	Adolescents (*N* = 174)	
School transport	Car		463 (62.48%)	258 (58.5%)	205 (68.33%)	203 (84.58%)	55 (83.33%)	148 (85.06%)	
	Bus		152 (20.51%)	106 (24.04%)	46 (15.33%)	37 (15.42%)	11 (16.67%)	26 (14.94%)	
	Foot		126 (17%)	77 (17.46%)	49 (16.33%)	0	0	0	
	Gender		Pearson Chi-Square = 56.296						.001
	Age stage		Pearson Chi-Square = 18.249						.001
	Within groups	Boys	Pearson Chi-Square = 9.487						.009
		Girls	Pearson Chi-Squar*e* = 0.109						NS
Breakfast on weekday (time/5 days)	0		18 (2.43%)	14 (3.17%)	4 (1.33%)	16 (6.67%)	3 (4.55%)	13 (7.47%)	
	1		141 (19.03%)	84 (19.05%)	57 (19%)	36 (15%)	14 (21.21%)	22 (12.64%)	
	2		170 (22.94%)	107 (24.26%)	63 (21%)	27 (11.25%)	6 (9.09%)	21 (12.07%)	
	3		111 (14.98%)	61 (13.83%)	50 (16.67%)	28 (11.67%)	8 (12.12%)	20 (11.49%)	
	4		72 (9.72%)	43 (9.75%)	29 (9.67%)	43 (17.92%)	9 (13.64%)	34 (19.54%)	
	5		229 (30.9%)	132 (29.93%)	97 (32.33%)	90 (37.5%)	26 (39.4%)	64 (36.78%)	
	Gender		Pearson Chi-Square = 37.592						.001
	Age stage		Pearson Chi-Square = 6.293						NS
	Within groups	Boys	Pearson Chi-Square = 4.613						NS
		Girls	Pearson Chi-Square = 4.368						NS
Breakfast on weekend (times/2 days)	0		155 (20.92%)	96 (21.77%)	59 (19.67%)	46 (19.17%)	15 (22.73%)	31 (17.82%)	
	1		289 (39%)	170 (38.55%)	119 (39.67%)	63 (26.25%)	16 (24.24%)	47 (27.01%)	
	2		299 (40.35%)	175 (39.68%)	122 (40.67%)	131 (54.58%)	35 (53.03%)	96 (55.17%)	
	Gender		Pearson Chi-Square = 16.886						.001
	Age stage		Pearson Chi-Square = 10.63						NS
	Within groups	Boys	Pearson Chi-Square = 0.134						NS
		Girls	Pearson Chi-Square = 0,786						NS
Sleep quality (participants)	Some bad time		325 (43.86%)	176 (39.91%)	149 (49.67%)	95 (39.58%)	13 (19.7%)	82 (47.13%)	
	Trouble waking		210 (28.34%)	88 (19.95%)	122 (40.67%)	30 (12.5%)	7 (10.61%)	23 (13.22%)	
	Gender		Pearson Chi-Square = 10.188						.002
	Age stage		Pearson Chi-Square = 1.829						NS
	Within groups	Boys	Pearson Chi-Square = 2.044						NS
		Girls	Pearson Chi-Square = 1.787						NS

Table 3 presents the sleep durations on weekdays and weekends, as well as the PA and sedentary behaviour scores of the participants, stratified by sex and age. Boys slept more on weekdays (488.90 ± 74.33 vs. 467.76 ± 78.75 min. night ^−1^), were more active (scores: 2.58 ± 0.69 vs. 2.34 ± 0.82), used laptops more frequently (scores: 2.46 ± 1.51 vs. 1.90 ± 1.63) and played more video games (scores: 3.12 ± 1.43 vs. 1.2875 ± 1.36) than girls. Girls were less sedentary than boys (scores: 2.63 ± 0.76 vs. 2.9±.79). However, girls watched more television (scores: 3.65 ± 1.155 vs. 2.73 ± 1.48) and used their smart phones more frequently (scores: 3.6625 ± 1.3 vs. 3.28 ± 1.44) significantly more (*p* < .001 overall). The ANOVA test further showed that prepubescent boys spent more time in bed during the week than the two groups of adolescents (*p* < .01, boys; *p* < .001, girls), as did the adolescent boys compared to the two groups of girls (*p* < .01 both). A significant difference was also found in weekday sleep duration between adolescent boys and girls (*p* < .001). Adolescent boys were significantly more physically active than the other three groups (*p* < .001 for all), but adolescent girls were least sedentary (*p* < .001 for prepubescent boys; *p* < .01 for prepubescent girls). In total, 62 prepubescent boys (14.06%), 130 adolescent boys (43.33%), 23 prepubescent girls (34.85%), and 38 adolescent girls (21.845%) were classified as active (PA score equal to 3 or more).

**Table 3.** Sleep duration on weekdays and weekends, physical activity levels, and sedentary behaviours of students in private schools in the Al-Ahsa governorate, Saudi Arabia, stratified by sex and age.

		Sleep duration on weekday (min./night)	Sleep duration on weekend (min./night)	PA score	Sedentary behaviours score	Watching TV score	Laptop use score	Video games use score	Smartphone use score
Between groups	F	20.545	2.383	56.737	12.054	34.878	31.910	102.925	13.213
	Sig.	.000	.068	0.001	0.001	0.001	0.001	0.001	0.001
Boys	Total (*N* = 741)	488.90 ± 74.33***	587.59 ± 123.08	2.58 ± 0.69 ***	2.9±.79 ***	2.73 ± 1.48 ***	2.46 ± 1.51 ***	3.12 ± 1.43 ***	3.28 ± 1.44 ***
	Children (*N* = 441)	497.02 ± 76.24 b, d	595.80 ± 131.82	2.33 ± 0.57 b	2.92 ± 0.8 d	2.95 ± 1.44 b, c, d	2.57 ± 1.45 c, d	3.08 ± 1.45 c, d	3.09 ± 1.42 b, d
	Adolescents (*N* = 300)	476.98 ± 69.86 a, c, d	575.52 ± 108.05	2.94 ± 0.7 a, c, d	2.86 ± 0.78 d	2.42 ± 1.49 a, c, d	2.31 ± 1.58 c, d	3.17 ± 1.41 c, d	3.57 ± 1.43 a
Girls	Total (*N* = 240)	467.76 ± 78.75	580.32 ± 115.08	2.34 ± 0.82	2.63 ± 0.76	3.65 ± 1.155	1.90 ± 1.63	1.2875 ± 1.36	3.6625 ± 1.3
	Children (*N* = 66)	512.55 ± 55.23 b, d	595,68 ± 106.38	2.38 ± 0.98 b	2.91 ± 0.9 d	3.59 ± 1.27 a, b	3.15 ± 1.60 a, b, d	1.46 ± 1.38 a, b	3.47 ± 1.44
	Adolescents (*N* = 174)	450.77 ± 79.79 a, b, c	574.49 ± 117.99	2.32 ± 0.75 b	2.51 ± 0.69 a, b, c	3.67 ± 1.11 a, b	1.42 ± 1.38 a, b, c	1.17 ± 1.31 a, b	3.79 ± 1.2 a

Note:

Data are means ± standard deviation. a, b, c, d differs for prepubescent boys, adolescent boys, prepubescent girls, and adolescent girls, respectively, as determined by ANOVA analysis. *** < .001 differs to girls using *t*-test for independent samples. PA: physical activity.

Stepwise multiple regression analysis revealed that BMI was negatively associated with PA score and positively associated with sedentary behaviours in the overall group and in all sex-age stratified subgroups, except for sedentary behaviours in prepubescent girls (Table 4). Playing video games and using laptops were sedentary activities significantly associated with BMI in the overall group (video game: *β* = 0.593, *p* < .001; laptops: *β* = 0.238, *p* < .01). However, in the subgroups, significance was detected in prepubescent boys for video games only (*β* = 0.869; *p* < .001) and in adolescent boys for laptop use only (*β* = 0.489; *p* < .001). Breakfast frequency also significantly affected BMI in the overall group (*β* = 0.214; *p* < .01), as well as in adolescent boys (*β* = 0.366; *p* < .01) and adolescent girls (*β* = 0.227; *p* < .05). BMI was further associated with school transportation among prepubescent girls (*β* = −2.138; *p* < .01) and weekend sleep duration in adolescent girls (*β* = −0.004; *p* < .05).

**Table 4.** Stepwise multiple regression analysis models for the associations between BMI and school transportation, breakfast frequency, sleep patterns, daily physical activity, and sedentary behaviours among students in private schools in the Al-Ahsa governorate, Saudi Arabia, stratified by sex and age.

Model 1					Model 2				
Variables		*R* ^2^	*β* coef. (SE)	*t*	Variables	*R* ^2^	*β* coef. (SE)	*t*	
Total (*N* = 981)	Constant	0.184	20.076 (0.709) ***	28.296	Constant	0.192	21.523 (0.587) ***	36.677	
	PA score		−1.978 (0.172) ***	−11.471	PA score		−2.01 (0.171) ***	−11.727	
	Sedentary behaviours score		1.22 (0.159) ***	7.652	Playing video games		0.566 (0.079) ***	7.145	
	Breakfast frequency		0.222 (0.077) **	2.872	Breakfast frequency		0.246 (0.077) **	3.185	
					Using laptops		0.218 (0.082) **	2.912	
Prepubescent boys (*N* = 441)	Constant	0.164	22.076 (1.361) ***	18.313	Constant	0.196	22.022 (1.039) ***	21.196	
	PA score		−2.746 (0.360) ***	−7.620	PA score		−2.470 (0.359) ***	−6.885	
	Sedentary behaviours score		1.118 (0.257) ***	4.342	Playing video games		0.869 (0.142) ***	6.114	
Adolescent boys (*N* = 300)	Constant	0.330	25.275 (2.151) ***	18.566	Constant	0.329	27.203 (1.117) ***	24.343	
	PA score		−3.271 (0.314) ***	−10.425	PA score		−3.32 (0.313) ***	−10.599	
	Sedentary behaviours score		1.048 (0.28) ***	3.746	Using laptops		0.51 (0.139) ***	3.677	
	Breakfast frequency		0.417 (0.139) **	2.993	Breakfast frequency		0.43 (0.139) **	3.090	
Prepubescent girls (*N* = 66)	Constant	0.446	25.258 (1.065) ***	23.716	Constant	0.446	25.258 (1.065) ***	23.716	
	PA score		−1.701 (0.254) ***	−6.683	PA score		−1.701 (0.254) ***	−6.683	
	Transport		−2.138 (0.665) **	−3.217	Transport		−2.138 (0.665) **	−3.217	
Adolescent girls (*N* = 174)	Constant	0.462	20.058 (1.413) ***	14.196	Constant	0.448	24.586 (1.173) ***	20.962	
	PA score		−1.933 (0.261) ***	−7.399	PA score		−2.062 (0.261) ***	−7.911	
	Sedentary behaviours score		1.974 (0.286) ***	6.905	Using laptops		1.020 (0.141) ***	7.215	
	Sleep on weekend		−0.004 (0.002) *	−2.512	Sleep on weekend		−0.004 (0.002) ***	−2.447	
	Breakfast frequency		0.227 (0.114) *	1.985					

Note: Note: Regression *β* coefficients (unstandardised) represent the degree of change in the BMI for every 1-unit of change in the predictor variable. Model 1 performed with sedentary behaviour scores and Model 2 with sedentary activity levels. *p < .05, ** *p* < .01, ***p < .001. PA: physical activity; SE: standard error.

Table 5 shows the sleep duration, PA, and sedentary behaviour scores of male participants, stratified by age and BMI. No significant differences existed in sleep duration, watching television, and using smartphones between all BMI subgroups of children, or sleep duration, playing video games, or using smartphones between all BMI subgroups of adolescents. However, both obese children and adolescents were significantly less active (*p* < .001 for all) and more sedentary (*p* < .01, UW and OW, respectively; *p* < .001 for the rest) than their healthy peers. Significantly lower laptop use was also noted in children in the OW group compared to their NW and OB peers (*p* < .01, for both), as well as video games in children in the UW, NW, and OW groups compared to children in the OB group (*p* < .001 for all). In addition, a significantly low laptop use score, and short sedentary television viewing time, were found in adolescents in the NW group compared to the OB group (*p* < .001 for both).

**Table 5.** Sleep duration on weekdays and weekends, physical activity levels and sedentary behaviours of male students in private schools in the Al-Ahsa governorate, Saudi Arabia, stratified by sex, age, and BMI.

				Boys									
	Children (*N* = 441)					Between BMI-groups		Adolescents (*N* = 300)				Between BMI-groups	
	UW	NW	OW		OB			UW	NW	OW	OB	*F*	*P* value
	(*N* = 49)	(*N* = 211)	(*N* = 62)		(*N* = 119)	*F*	*P* value	(*N* = 46)	(*N* = 154)	(*N* = 33)	(*N* = 67)		
Sleep weekdays (min./day)	501.29	497.92	502.11		491.00	0.399	.754	494.43	477.73	476.21	463.63	1.795	.148
	(66.38)	(69)	(69.26)		(94.17)			(61.64)	(67.72)	(70.66)	(77.94)		
Sleep weekend days (min./day)	590.20	595.88	601.00		595.24	0.062	.980	553.15	573.93	609.09	577.99	1.755	.156
	(109.07)	(142.80)	(140.92)		(115.63)			(63.15)	(99.57)	(143.60)	(127.51)		
PA score	2.66	2.42	2.47		1.95	32.07	.001	3.18	3.18	2.87	2.27	39.147	.001
	(0.56) d	(0.52) d	(0.60) d		(0.45) a, b, c			(0.41) d	(0.63) d	(0.59) d	(0.62) a, b, c		
Sedentary behaviours score	2.83	2.8	2.74		3.26	10.336	.001	2.76	2.74	2.69	3.32	10.502	.001
	(0.92)d	(0.79)d	(0.67)d		(0.73) a, b, c			(0.83) d	(0.71) d	(0.69) d	(0.82) a, b, c		
Watching TV score	2.98	2.85	2.97		3.09	0.741	.528	2.46	2.09	2.33	3.18	9.032	.001
	(1.56)	(1.39)	(1.57)		(1.44)			(1.46)	(1.43) d	(1.51)	(1.38) b		
Laptop use score	2.37	2.69	2.00		2.73	4.595	.004	2.24	2.07	2.06	3.01	6.255	.001
	(1.67)	(1.39)c	(1.27)b, d		(1.49)c			(1.42)	(1.48)d	(1.41)	(1.78)b		
Video games use score	2.55	2.75	3.03		3.90	21.155	.001	2.78	3.21	2.97	3.45	2.322	.075
	(1.39)d	(1.36)d	(1.58)d		(1.22)a, b, d			(1.35)	(1.39)	(1.31)	(1.49)		
Smartphone use score	3.43	2.94	2.95		3.30	2.857	.037	3.54	3.58	3.39	3.63	.211	.889
	(1.29)	(1.44)	(1.45)		(1.37)			(1.49)	(1.37)	(1.46)	(1.54)		

Note: Data are means ± standard deviation. a, b, c, d differs to UW, NW, OW, and OB in the same age group, respectively, as determined by ANOVA analysis. PA: physical activity; UW: underweight; NW: normal weight; OW: overweight; OB: obese.

For girls (Table 6), significant differences were observed in daily PA scores between children in the UW group and their OW and OB peers (*p* < .01 all). Important, but statistically non-significant differences were noted in the PA scores of children in the NW group compared to the OW (2.52 ± 0.97 vs. 1.73 ± 0.43) and OB (2.52 ± 0.97 vs. 1.35 ± 0.34) groups. No significant differences were observed in the sedentary behaviours between all BMI groups of children, despite those found in sedentary television viewing time between the OW and OB groups compared with the UW and NW groups (*p* < .01 all). Obese adolescent girls were less active and more sedentary than their UW and NW peers (all *p* < .001). Moreover, they used laptops (*p* < .001) and video game consoles (*p* < .001) more frequently. A significant difference was also recorded in the UW group compared to the OW group of adolescent girls in the PA score (*p* < .01), as well as in the UW and NW groups compared to the OW group in sedentary behaviour scores (*p* < .001 for both) and playing video games (*p* < .001 for both). No significant differences were found in sleep duration, watching television, or using smartphones between groups of adolescent girls stratified by BMI.

**Table 6.** Sleep duration on weekdays and weekends, physical activity levels, and sedentary behaviours of female students in private schools in the Al-Ahsa governorate, Saudi Arabia, stratified by sex, age, and BMI.

				Girls									
	Children (*N* = 66)					Between BMI-groups		Adolescents (*N* = 174)				Between BMI-groups	
	UW (*N* = 7)	NW (*N* = 43)	OW (*N* = 12)		OB (*N* = 4)	*F*	*P* value	UW (*N* = 25)	NW (*N* = 127)	OW (*N* = 13)	OB (*N* = 9)	*F*	*P* value
Sleep weekdays (min./day)	539.29	503.79	525.83		520.00	2.417	.490	467.28	449.24	431.46	454.44	0.628	.598
	(86.72)	(55.18)	(34.17)		(24.15)			(80.59)	(77.52)	(97.81)	(87.62)		
Sleep weekend days (min./day)	587.14	590.00	610.00		628.75	0.884	.829	636.76	566.60	528.08	580.00	3.316	.021
	(48.55)	(123.26)	(47.19)		(129.25)			(127.80)	(114.97)	(85.67)	(127.28)		
PA score	3.22	2.52	1.73		1.35	16.193	.001	2.78	2.34	2.01	1.23	12.202	.001
	(0.97)c, d	(0.97)	(0.43)a		(0.34)a			(0.57)c, d	(0.70)d	(0.70)a	(0.64)a, b		
Sedentary behaviours score	3.00	2.76	3.31		3.63	2.461	.071	2.35	2.39	3.27	3.69	21.350	.001
	(0.68)	(0.86)	(0.81)		(0.25)			(0.63)c, d	(0.57)c, d	(0.77) a, b	(0.46) a, b		
Watching TV score	2.71	3.44	4.25		4.75	11.57	.009	3.44	3.59	4.31	4.44	3.705	.013
	(0.95)c, d	(1.2)c, d	(0.97)a, b		(0.50)a, b			(1.12)	(1.08)	(1.03)	(0.88)		
Laptop use score	3.43	2.91	3.67		3.75	2.473	.480	1.36	1.17	2.31	4.00	17.783	.001
	(1.9)	(1.67)	(1.23)		(0.96)			(1.47) d	(1.10) d	(1.70)	(1.12) a, b		
Video games use score	1.57	1.32	1.83		1.5	0.461	.071	0.92	0.92	3.15	2.56	20.150	.001
	(1.27)	(1.39)	(1.34)		(1)			(1.12)c, d	(1.09)c, d	(1.34)a, b	(1.51) a, b		
Smartphone use score	3.71	3.39	3.5		3.75	0.152	.928	3.64	3.87	3.31	3.78	1.041	.376
	(1.38)	(1.48)	(1.45)		(1.5)			(1.04)	(1.19)	(1.49)	(1.30)		

Note:

Data are means ± standard deviation. a, b, c, d differs from UW, NW, OW and OB in the same age group, respectively, as determined by ANOVA analysis. PA: physical activity; UW: underweight; NW: normal weight; OW: overweight; OB: obese.

## 4. Discussion

This study examined the effects of breakfast intake, duration and quality of sleep, daily PA, and sedentary behaviours on the BMI of private school students, aged 10–15 years, in the Al-Ahsa governorate, in the eastern province of Saudi Arabia. The overall prevalence of overweight and obesity was 12.82% and 25.1% in boys, and 10.42% and 5.42% in girls, respectively. Most participants used cars or buses as transportation to and from school (100% of girls and 83% of boys). Breakfast-skipping was significant, especially among male participants. Boys slept more than girls on school days, and they were more active, used laptops more frequently, and played more video games than girls. Girls were less sedentary, watched television more often, and used smartphones more frequently than boys did. Stepwise multiple regression analysis revealed significant associations between BMI and breakfast intake, PA, and sedentary behaviours, of which using laptops and playing video games were the key sedentary activities that were most associated with BMI.

The results showed several differences from those recorded nationally and in other cities. The WHO [17] reported that among Saudi youths, aged 5–19 years, 38.3% of boys and 32.3% of girls were overweight, and 19% of boys and 13.6% of girls were obese. However, Aliss et al. [18] noted that approximately 40% of boys and 10% of girls, aged 5–15 years, were overweight and obese in the Jeddah region in the west of Saudi Arabia. Farsi and El-Khodary [19], and El-Khodary and Farsi [20] also reported that almost 40% of boys and girls are overweight and obese in the Jeddah region, with 18% of children and 16% of adolescents being overweight [19]. However, Al-Hussaini et al. [21] reported a prevalence of overweight and obesity of 13.4% (14.2% for girls; 12% for boys) and 18.2% (18% for girls; 18.4% for boys), respectively, among 7,930 schoolchildren, aged 6–16 years, in the central region city of Riyadh. Unfortunately, studies conducted in other regions of Saudi Arabia are relatively dated. They reported an average prevalence of overweight and obesity of 26% among adolescents in the Asir and Hail regions, less urbanised towns in northwestern Saudi Arabia [22], and an average prevalence of overweight and obesity of 19.0% and 23.3%, respectively, in the Eastern Province [23].

Eating breakfast showed a significant positive association with BMI, which was more significant in boys than in girls, and in children than in adolescents. Our results contradicted those of Szajewska and Ruszczynski [24], who demonstrated in a systematic review of 16 European studies that breakfast consumption was negatively associated with BMI and the risk of becoming overweight or obese in children and adolescents. Although identifying the factors responsible for this discrepancy is difficult, it is possible that eating breakfast with low nutritional value, such as eating out and consuming foods excessively high in fat, calorie density, and refined carbohydrates, is widely observed among the Saudi population in recent decades, is responsible for the positive association between BMI and breakfast consumption [25,26].

Daily breakfast eating rates were 35.63% (30.9% on weekdays and 40.35% on weekends) in boys and 46.04% (37.5% on weekdays and 54.58% on weekends) in girls. These findings are higher than those reported by Al-Hazzaa et al. [27] in 2,908 Saudi adolescents from Riyadh, Jeddah, and Al-Khobar cities (28.7% of boys and 20.6% of girls). However, breakfast-skipping rates in our population remain higher than the estimated 10–30% reported worldwide [28]. The health benefits of breakfast for children have been proven. Yet, studies on breakfast intake and associated behaviours in Saudi schoolchildren and adolescents are still limited, and available data are disparate [25]. Comparing the prevalence of breakfast intake between children attending public and private elementary schools in the city of Jeddah, western Saudi Arabia, Jabri et al. [29] noted similar rates between both groups (20.6% vs. 19.4%), with significantly higher daily breakfast intake in boys (26.3%) than girls (13.3%) in private schools. The proportion of daily breakfast intake was also almost 21% in public and private schools in Riyadh city, central Saudi Arabia, with no significant difference between boys and girls (19.3% vs. 22.1%). Nonetheless, daily breakfast intake on weekends (41.3%) was significantly greater than on weekdays (28.9%) in girls versus boys in public schools (13.3% vs. 21.4%), as well as in boys in private schools versus boys in public schools (32.5% vs. 13.3%) [26]. These studies also indicated that to achieve nutritional goals and improve well-being, not only is breakfast consumption important, but also the nutritional composition of breakfast. For example, regular consumption of cereals for breakfast has been shown to lower the BMI and decrease the risk of being overweight or obese. It is also more likely to meet the recommended intake of B vitamins, calcium, iron, and fibre without increasing the total energy intake or sodium content [28]. Unfortunately, our study did not examine the composition of breakfast and its associated factors. Therefore, complementing this study by evaluating breakfast consumption patterns and variables associated with daily breakfast consumption in the future could provide beneficial information.

Another interesting and expected finding from this study was the higher negative association between overweight and obesity and the daily PA score. Our results corroborate with the findings from the WHO [30], that classified energy intake and PA as the most convincing factors for promoting a healthy weight. According to Laughlin et al. [31], the altered energy efficiency of moderate and continuous PA appears to be the dominant contributor to metabolic adjustment with weight loss. PA increases the efficiency of muscle mitochondria; thus, mitochondria produce more energy per unit of oxygen consumed. Other specific mechanisms may also contribute to energy efficiencies, such as improved concentration, sensitivity to hormones, and changes in muscle contractile proteins and thermoregulatory processes including futile cycles, activation of brown fat, thyroid hormone, skin thickness, and vasodilation [31].

Our results also revealed that for each 1-unit increase in daily PA score, BMI decreased by nearly 2.746 kg.*m*^−2^ in prepubescent boys, 3.318 kg.*m*^−2^ in adolescent boys, 1.701 kg.*m*^−2^ in prepubescent girls, and 1.933 kg.*m*^−2^ in adolescent girls. Additionally, boys were more active than girls and adolescent boys than the other three groups, stratified by sex and age. Significant differences in PA scores were observed in different age groups of obese boys compared to the other BMI groups of boys, in underweight prepubescent girls compared to their overweight or obese peers, and in obese adolescent girls compared to their underweight or normal weight peers. Our observations are consistent with those of Aliss et al. [18]. In their study of 118 boys and 82 girls, aged 5–15 years, in the Jeddah region, more than half the boys (59%) and more than a third of the girls (40%) were classified as very active, with a higher proportion of girls classified as having a low activity (38% vs. 26%). Contrary to our results, that study found that, regardless of sex, children were more active than adolescents (40% vs. 21% for girls; 64% vs. 38% for boys). Likewise, Al-Nuaim et al. [32] reported a significant negative association between PA and BMI in boys only among 1,270 students (boys 52%, girls 48%), aged 15–19 years in the eastern region of Saudi Arabia [33]. Using an electronic pedometer, Al-Hazzaa [33] counted an average of 13,489 ± 5,791 steps per day among 296 obese and non-obese schoolchildren aged 8–12 years, in Riyadh area. The study also noted that obese boys showed significantly lower body fat percentage and BMI and were significantly less active (10,602 ± 4,800 steps/day) than boys who were not obese (14,271 ± 5,576 steps/day).

Any comparison between studies on PA should be made with caution because large variations between studies can exist, in the characteristics of participants, as well as the methods and techniques used in the evaluation process. Nevertheless, in a systematic review of 42 studies conducted at the national, subnational, and local levels, Al-Hazzaa [34] claimed that most Saudi children and youths did not meet the minimum weekly PA recommended by international guidelines. The current Physical Activity Guidelines [5] suggest that children, aged 6–17 years, should engage in moderate to vigorous PA for at least 60 min on most days [35]. According to Yang [36], light physical intensity refers to an exercise intensity of less than 3 metabolic equivalents (METs), moderate physical intensity corresponds to 3 − 5.9 METs, and high intensity corresponds to 6 or more METs. 1 MET corresponds to an energy expenditure of 1 kcal/kg/h for an adult at rest, which corresponds to the oxygen consumption of 3.5 ml/kg/min. Most people can sing during light-intensity activities, speak but not sing during moderate-intensity activities, but during high-intensity activities, even speaking can become difficult.

PA levels among young Saudis ranged from moderate among boys (mean prevalence 55.5%) to very low among girls (mean prevalence 21.9%), with the highest prevalence in the northern and central regions [32]. PA was also positively associated with sex (boys), self-efficacy, parenthood, school sports, and support from friends and family. Another factor that has also been widely considered as a cause of reduced engagement in PA among Saudi youths is the Saharan climate of Saudi Arabia, which is extremely hot in summer and extremely cold and windy in winter. Certain traditions combined with a lack of space and equipment necessary for PA, especially in schools, could also contribute to increased physical inactivity and a greater likelihood of obesity in students [22,34].

Our results also demonstrated a significant positive association between BMI and sedentary behaviours, namely, the use of laptops and video game consoles. It has been argued that sedentary behaviours are not simply a lack of PA but a set of individual behaviours in which the individual’s energy expenditure does not significantly increase beyond rest levels, such as using electronic devices (televisions, computers, tablets, and phones), driving, and reading. It is any waking behaviour characterised by an energy expenditure of ≤1.5 METs regardless of posture (sitting, reclining, or supine) [36].

Saudi Arabia has experienced considerable socio-economic development over the past four decades. This has been accompanied by increased use of cars and electronic devices in all facets of Saudi lives, leading to a significant increase in sedentary behaviours and physical inactivity, mainly among children and adolescents. Bahathig et al. [7] noted that approximately 92.7% of 399 healthy adolescent female students, aged 13–14 years, in Arar, northern Saudi Arabia, did not meet the recommended 60 min per day of moderate to vigorous PA. However, the overall average time spent in sedentary activities was 357.64 ± 86.29 and 470.51 ± 147.64 min per day on weekdays and weekends, respectively. Al-Hazzaa [34] highlighted in a brief observation of the results of the Arab Adolescent Lifestyle Study (ATLS) that 84% of boys and over 91% of girls spend more than 2 h per day in sedentary activities (screen time). Contrary to our findings, Al-Hazzaa noted that women consistently appeared to be at a greater risk than men with physical inactivity and sedentary behaviours. Recent evidence has emphasised that elevated levels of sedentary behaviour in children and adolescents have adverse effects on cardiovascular disease risk factors and overall survival regardless of other factors, including body weight, diet, and PA [35]. Lee et al. [37] noted that due to the excessive incidence of sedentary behaviours and reduced engagement in PA, the Saudi population was ranked among the populations most affected by non-communicable diseases related to sedentary behaviours and PA levels, including coronary heart disease, type 2 diabetes, positive cancers, and overall mortality.

### 4.1. Strengths and limitations

The strength of this study lies mainly in the large research sample that represented all students, aged 10–15 years, from Al-Ahsa private schools. The inclusion of many girls from a region known for its conservative customs and traditions, with adjustment of the data for age, sex, and BMI, is also a strength. Nevertheless, our findings should be interpreted with consideration to study design limitations. First, except for anthropometric data, the values recorded and analysed were based only on self-reported responses to a student questionnaire. Therefore, the possibility of recall bias and social desirability outcomes cannot be ruled out. Recall bias occurs when contributors forget certain events, values, or frequencies. However, asking students about usual or common events, as well as recording the number of breakfasts eaten, sleep duration, or participation in sports or video games in the past seven days, may have reduced the risk of recall bias. Second, the questionnaire was used as an indirect method to assess PA levels and sedentary behaviours. The use of direct measurement methods, such as pedometers or motion sensors, can provide much higher accuracy. Third, apart from the number of times breakfast was consumed, no information was evaluated regarding the quality and quantity of the food consumed, which are two important measures in determining the effects of breakfast on health. Finally, unlike boys, physical education has just been introduced into the education system for girls, and it is not yet implemented in all schools. This could be favourable for boys whose physical education is an integral part of their weekly school routines. Therefore, the absolute PA levels in this study should be interpreted with caution.

## 5. Conclusions

Referring to the sex-specific BMI-for-age percentile charts, the overall prevalence of overweight and obesity among students, aged 10–15 years, in private schools in the Al-Ahsa governorate was 12.82% and 25.1% in boys and 10.42% and 5.42% in girls, respectively. Additionally, a significantly higher prevalence of overweight and obesity was detected among prepubescent boys than in prepubescent girls. Our results differed from earlier findings on the prevalence of overweight and obesity nationwide and in other cities in the country. Breakfast intake, PA, and sedentary behaviours were the most common factors associated with BMI. All the girls used cars or buses for school transportation, and 67.08% of them ate breakfast three or more times per week. Among the boys, 17% walked to school and the rest used cars or buses. Boys were also less likely to eat breakfast before school, with 59.38% eating breakfast three times or less per week. The adolescent boys were the most physically active, while the adolescent girl group was the least sedentary. Both groups of boys were characterised by excessive use of laptops and video game consoles, whereas girls watched more television and used their smart phones more frequently. Specifically, the two groups of obese boys were significantly less active and more sedentary than the other BMI groups. The overweight prepubescent boys used laptops more than boys in the NW and OB groups, and the obese prepubescent boys played video games more than those in the other three BMI groups. Obese adolescent boys used laptops more frequently and watched television more than their peers. For girls, significant differences were observed in television viewing time in the overweight and obese groups compared to the other two BMI groups. Obese adolescent girls were less active, more sedentary, and used laptops and video game consoles more than their underweight or normal weight peers. Significant differences were also recorded between the UW and NW groups of adolescent girls and those in the OW group in terms of PA, sedentary behaviours, and video games, as well as those in the NW versus OW group in terms of sedentary behaviours and playing video games.

## Data Availability

All datasets used and/or analysed during the current study are available from the corresponding author upon reasonable request.
